# Gender differences in the association between adverse events in childhood or adolescence and the risk of premature mortality

**DOI:** 10.1038/s41598-022-23443-y

**Published:** 2022-11-09

**Authors:** Aline Fernanda de Souza, Roberta de Oliveira Máximo, Dayane Capra de Oliveira, Paula Camila Ramírez, Mariane Marques Luiz, Maicon Luis Bicigo Delinocente, Jair Licio Ferreira Santos, Andrew Steptoe, Cesar de Oliveira, Tiago da Silva Alexandre

**Affiliations:** 1 Graduate Program in Physical Therapy, Federal University of São Carlos, São Carlos, Brazil; 2 Escuela de Fisioterapia, Universidad Industrial de Santander, Bucaramanga, Colombia; 3 Graduate Program in Gerontology, Federal University of São Carlos, São Carlos, Brazil; 4 Department of Social Medicine, University of São Paulo, Ribeirão Preto, Brazil; 5 Department of Epidemiology and Public Health, University College London, London, UK; 6 Department of Gerontology, Federal University of São Carlos, Rodovia Washington Luís, Km 235, Sao Carlos, 13565–905 Brazil

**Keywords:** Geriatrics, Public health

## Abstract

To examine, by gender, the relationship between adverse events in childhood or adolescence and the increased risk of early mortality (before 80 years). The study sample included 941 participants of the English Longitudinal Study of Aging who died between 2007 and 2018. Data on socioeconomic status, infectious diseases, and parental stress in childhood or adolescence were collected at baseline (2006). Logistic regression models were adjusted by socioeconomic, behavioral and clinical variables. Having lived with only one parent (OR 3.79; *p* = 0.01), overprotection from the father (OR 1.12; *p* = 0.04) and having had an infectious disease in childhood or adolescence (OR 2.05; *p* = 0.01) were risk factors for mortality before the age of 80 in men. In women, overprotection from the father (OR 1.22; *p* < 0.01) was the only risk factor for mortality before the age of 80, whereas a low occupation of the head of the family (OR 0.58; *p* = 0.04) and greater care from the mother in childhood or adolescence (OR 0.86; *p* = 0.03) were protective factors. Independently of one’s current characteristics, having worse socioeconomic status and health in childhood or adolescence increased the risk of early mortality in men. Parental overprotection increased the risk of early mortality in both sexes, whereas maternal care favored longevity in women.

## Introduction

Social determinants of health have received considerable attention as a fundamental principle in the field of public health^1^. The World Health Organization defines these determinants as conditions or circumstances in which people are born, grow, live, work and grow old^1^. Adverse experiences in childhood and adolescence, such as physical, sexual or emotional abuse, problems in the family environment (parents who used drugs and/or alcohol, exposure of the children to violence or illegal activities on the part of the parents and disintegration of the family nucleus) are considered important factors that can influence the course of life^2^. Furthermore, early conditions, which are defined as a heterogeneous set of factors that encompass intrauterine nutritional status, exposure to infectious and parasitic diseases, stressful environments (negligence and/or overprotection) and socioeconomic status in early life^3^ (characterized as adverse events experienced in childhood or adolescence and the focus of the present study), also seem capable of influence on the course of life.

Exposure to these events in early life may cause biological changes, including epigenetic mechanisms capable of altering neural structure and function during the growth phase, which in turn may lead to the adoption of unhealthy habits^4^. Moreover, such biological changes lead to a cascade of physiological processes capable of accelerating the development of chronic diseases in the long term and increasing the risk of premature death^4^.

There is evidence that living in stressful environments in the early years of life, with bad parental relationships, increases the risk of premature death^5^. The mechanism that may explain this increased risk of death is related to an increase in the prevalence of mental health problems and unhealthy lifestyle because of these adverse early life events. However, there seems to be gender differences with regards to individual responses to these adverse events in childhood or adolescence, that may affect longevity^6^. Women seem to be more likely to internalize negative emotions and, therefore, have a greater frequency of mental disorders, whereas men seem to have a greater proneness to alcoholism and drug use^7^.

Low socioeconomic status, both parental^8^ occupation and family structure^9^, may influence access to education, the quality of food offered and access to health services^8,9^. Consequently, a poor education in childhood or adolescence increases the likelihood of having a low-paying occupation in adulthood, whereas a scarcity of food and low access to health services in childhood or adolescence increase the risk of comorbidities in adulthood, which are highly associated with premature death^10^. However, the effect of living in an adverse socioeconomic situation in early life seems to be less significant for men, as they tend to be less dependent on the status of their family compared to women^11^.

Another important adverse event experienced in childhood or adolescence mediated by socioeconomic status is poor health in this period^3^. Often due to insufficient financial resources, a lack of hygiene in this phase has been related to repetitive cycles of infections of different types, such as respiratory^12^ and gastrointestinal^13^. Infections in childhood or adolescence have been linked to a greater frequency of chronic obstructive pulmonary disease and gastrointestinal diseases in adulthood and old age, independently of sex, with repercussions in the risk of death^12,14–16^.

Although the literature demonstrates adverse events in childhood or adolescence increase the prevalence of chronic diseases^2^, alcoholism and drug use^7^, smoking^17^ and a sedentary lifestyle^18^, all of which are risk factors of premature death, an important gap remains regarding the extent to which adverse events could increase the risk of premature death and whether the results are different between men and women. Understanding the effects of adverse events in childhood or adolescence could assist in the creation of public policies directed at ensuring better care for children and adolescents and, ultimately, the promotion of healthy aging. Therefore, the present study tests two hypotheses: (1) worse parental relationships, socioeconomic status and health events in childhood or adolescence can increase the risk of death before the age of 80; (2) there are gender differences in these associations.

## Methods

The English Longitudinal Study of Ageing (ELSA) is an ongoing panel study involving a representative sample of individuals aged 50 or older residing in England that began in 2002. The ELSA sample is composed of individuals who had previously participated in the Health Survey for England^19^ an annual health survey for which a different nationally representative sample is recruited every year using randomized, stratified, multi-stage, probabilistic sampling. Follow-up interviews for the ELSA study occur every two years and health examinations are preformed every four years. A detailed description of the study can be found elsewhere^20^.

The present longitudinal study used data from wave 3 of the ELSA study (2006–2007), which was the first wave that included questions on adverse events in childhood or adolescence. Among the 8810 individuals aged ≥ 50 years interviewed at baseline (2006/7), 1775 (20.1%) died during the 12 years of follow-up. Among the 1775 cases of deaths, 834 (46.9%) were excluded due to incomplete information on adverse events in childhood or adolescence and covariates in 2006. Thus, the final analytical sample of the present study was composed of 941 cases of death that occurred between 2006 and 2018 (445 women and 496 men). Differences between included/excluded subjects can be seen in the Supplemental Material.

### Mortality

The cases of death were classified into two groups: (a) deaths having occurred after the age of 80; and (b) deaths having occurred before the age of 80. The mortality data were obtained from the English mortality system.

### Adverse events in childhood or adolescence

**Figure 1:**
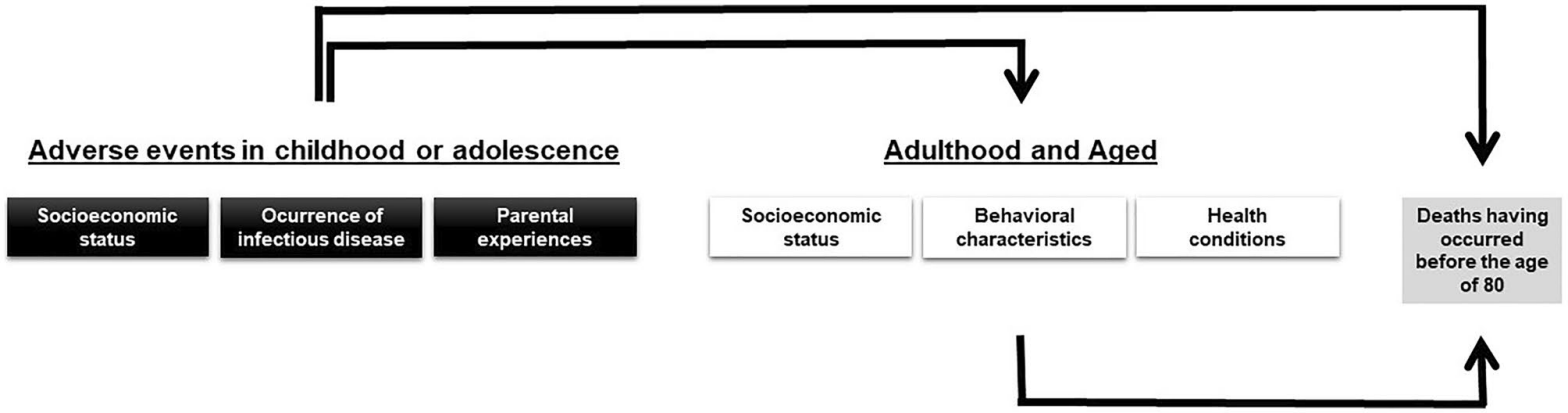
Hierarchical theoretical model of effects of variables of interest on mortality.

In the present study, adverse events in childhood or adolescence with the potential to influence mortality were divided into three major categories: socioeconomic status (family structure, occupation of head of the family and living conditions), the occurrence of infectious diseases and parental experiences (Fig. 1).

Parental experiences were assessed using the Parental Bonding Instrument (PBI), which is composed of questions addressing perceptions in two domains: protection and care from parents in childhood or adolescence^21^. Bad parenting characterized as low levels of care (negligence) and overprotection (absence of autonomy) are believed to be capable of triggering stress in childhood or adolescence with consequences throughout life^5^.

In the present study, we used the validated short version of the PBI^22^ composed of seven questions with scores ranging from 0 to 3 points. The care domain is composed of three questions [total score: 0 (less favorable) to 9 (more favorable) points] and the protection domain is composed of four questions [total score: 0 (more favorable) to 12 (less favorable) points]. The instrument was administered separately for the relationship with the father and the relationship with the mother, resulting in a score^23^ per domain classified as follows: care from father and care from mother (0 to 9 points each), protection from father and protection from mother (0 to 12 points each). The scores of each domain were considered discrete quantitative variables.

Infectious diseases in childhood or adolescence were based on reports of at least one of the following conditions: measles, mumps, poliomyelitis, tuberculosis, diphtheria, gastroenteritis, rubella, hepatitis, malaria, meningitis, pneumonia, scarlet fever, septicemia, and whooping cough^3^. The variable was classified as absent or present (absent = 0; present = 1).

Family structure in childhood is an indicator of the provision of resources that can influence education, health and socioeconomic status in adulthood^24^. Magnusson and Berger reported that family structure is an important aspect in the development of children through to adulthood. Adults who spend the initial cycle of life living with their biological parents would have, on average, a lower frequency of behavioral, academic and social problems throughout the entire life cycle^24^. Thus, in the present study, family structure was categorized as having lived with both parents (reference group = 0), having lived with the father and stepmother or mother and stepfather (coded 1), having lived with only the father or only the mother (coded 2) and having lived with other people (coded 3)^8^.

The occupation of the head of the family is an important indicator of socioeconomic status^8^. Besides being seen as a proxy of social prestige and an indicator of cultural aspects, occupational class has been described as a possible link between education and income; for a significant portion of the population, a better education translates to a better occupation, which ensures a higher income^25^. Occupational class of the head of the household was classified as "high" [e.g. managerial, entrepreneurial/administrative positions and specialized commerce (reference group = 0)] or "low" [e.g. manual labor, part time/temporary job, ill individuals and individuals with disability (coded 1)]^26^.

Housing conditions in childhood were also used as an important variable for the description of socioeconomic status^27^. The literature indicates that growing up under precarious housing conditions makes individuals susceptible to situations that can lead to health risks^27^. Thus, we evaluated the number of amenities in the home (e.g. hot and cold running water supply, central heating, inside toilet and access to a bath/shower inside the home). The final variable was categorized into having five amenities (reference group = 0) to having no amenities (coded 5). The number of rooms in the home, which is another proxy of the family financial situation in childhood used in studies on early living conditions^28^, was considered a discrete quantitative variable (greater number of rooms was considered more favorable). The number of residents in the home^29^, which was also considered a discrete quantitative variable (greater number of people was considered less favorable), was used as an indicator of crowding, which is indicated in the literature as something distinct from wealth^30^.

### Covariates

The covariates included at the baseline of the study (2006) were selected based on previous associations with adverse events in childhood or adolescence and mortality. The sociodemographic variables were race (white = 0 or non-white = 1), marital status (married = 0 or single = 1), educational years (> 13 years = 0; 12–13 years = 1; 0–11 years = 2) and total non-pension household wealth (the sum of financial wealth, the value of any home and other property [less mortgage], the value of any business assets and physical wealth owned by the household minus any debt) divided into quintiles (5th quintile [top 20%] = 0; 4th quintile = 1; 3rd quintile = 2; 2nd quintile = 3 and 1st quintile [lower 20%] = 4).

The behavioral characteristics analyzed were smoking (non-smoker = 0, ex-smoker = 1 or current smoker = 2) and alcohol intake (non-drinker or up to once per week = 0, two to six times per week = 1 and daily = 2)^31^. Level of physical activity was determined using the validated Health Survey for England questionnaire. Physical activity frequency was classified as more than once per week, once per week, one to three times per month and rarely or never. Intensity of physical activity was classified as mild (e.g. home repairs, vacuuming, washing clothes), moderate (e.g. dancing or stretching, washing the car, walking at a moderate pace, gardening) and vigorous (e.g. playing tennis, swimming, cycling, running, aerobic exercise, weightlifting or digging). The individuals were classified as having an active lifestyle (practice of mild, moderate or vigorous physical activity at least once per week [coded 0]) or sedentary lifestyle (practice of physical activity rarely or never, independently of intensity [coded 1])^32,33^.

Health conditions were evaluated based on self-reported doctor-diagnosed: stroke, heart disease, lung disease, cancer, osteoporosis, dementia, emotional/psychiatric problems, hypertension, diabetes mellitus and falls (no = 0; yes = 1). The presence of depression was assessed using the Center for Epidemiological Studies-Depression Scale (CES-D). For each CES-D question, participants answered “yes” or “no” and the scores ranged from 0 to 8, with 0 being not depressed and 8 being severely depressed^34^. The presence of depressive symptoms was defined as a score ≥ 4 points (coded 0: < 4 points; coded 1 ≥ 4 points)^35–37^.

Difficulty in performing basic activities of daily living (ADL) was evaluated using the modified Katz Index (e.g. bathing, feeding, walking, getting in or out of bed, dressing and using the toilet)^38,39^. Participants answered whether they did/did not have a difficulty performing each respective activity (no = 0; yes = 1). Difficulty in performing instrumental activities of daily living (IADL) was evaluated using the adapted Lawton Scale (e.g. housecleaning, washing clothes, preparing meals, using transportation, shopping, using the telephone, managing money and managing medications)^39,40^. Participants answered whether they did/did not have a difficulty performing each respective activity (no = 0; yes = 1). ADL and IADL were considered as discrete quantitative variables (greater number of difficulties was considered less favorable).

### Statistical analysis

The sample characteristics were expressed as means and proportions. Differences in the sample analyzed—women and men who died before and after 80 years of age—and those excluded due to missing information were determined using the chi-squared test and Student's t-test. Based on the literature, we performed an interaction analysis between the adverse events in childhood or adolescence variables and sex (women = 0; men = 1), which showed a significant difference (*p* < 0.05). Therefore, two logistic regression models (one for each sex) were created to analyze the associations between adverse events in childhood or adolescence and death before the age of 80 in the 12-year follow-up period. For the regression models, the covariates were selected based on their relationship with adverse events in childhood or adolescence or mortality. Variables with a *p*-value < 0.20 in the univariate analysis were selected for the multiple model and those with a *p*-value < 0.05 in the final model were considered significantly associated with the outcome^41^. All analyses were performed using Stata 14® (StataCorp, College Station, TX, USA).

### Ethical approval and informed consent

The London Multicentre Research and Ethics Committee (MREC 01/2/91) approved the ELSA study and the research was performed in accordance with relevant guidelines/regulations. All participants signed a statement of informed consent.

## Results

The mean age of individuals in the category who died after the age of 80 was 88.4 years for men and 87.1 years for women, while for those in the category who died before the age of 80, it was 73.9 years for men and 74.7 years for women. Overall, participants who died after the age of 80, compared with those who died earlier, consumed less alcohol, smoked less and reported a lower frequency of infectious diseases in childhood or adolescence (Tables 1 and 3).

**Table 1:** Baseline socioeconomic and behavioral characteristics who died before and after the age of 80 during 12-month follow-up.

	Full sample (N = 941)		Women (n = 445)		Men (n = 496)	
	Death before the age of 80 (n = 339)	Death after the age of 80 (n = 602)	Death before the age of 80 (n = 136)	Death after the age of 80 (n = 309)	Death before the age of 80 (n = 203)	Death after the age of 80 (n = 293)
**Socioeconomic aspects**						
Age (mean), (SD)	74.4 (4.2)*	87.8 (5.0)*	73.9 (4.3)^δ^	88.4 (4.9)^δ^	74.7 (4.1)^§^	87.1 (5.0)^§^
Race (non-white), %	0.9	0.5	0.7	0.6	1.0	0.3
Marital status (single), %	34.2*	52.2*	44.8^δ^	69.6^δ^	27.1	33.8
Educational years, %						
> 13 years	24.2	20.6	16.2	13.9	29.6	27.7
12–13 years	21.5	17.1	24.2	17.5	19.7	16.7
0–11 years	54.3	62.3	59.6	68.6	50.7	55.6
Total household wealth (quintiles), %						
5th quintile (top)	14.8	15.0	15.4	11.0	14.3	19.1
4th quintile	18.6	21.3	17.7	18.1	19.2	24.6
3rd quintile	19.2	22.6	16.9	22.3	20.7	22.9
2nd quintile	23.0	18.1	27.2	22.0	20.2	14.0
1st First quintile (lower)	23.3	21.9	22.1	25.3	24.1	18.4
Not declared	1.1	1.1	0.7	1.3	1.5	1.0
**Health behaviors**						
Smoking, %						
Non-smoker	25.1*	35.2*	36.8^δ^	46.9^δ^	17.2^§^	22.9^§^
Ex-smoker	47.8	55.5	35.3	43.0	56.2^§^	68.6^§^
Smoker	27.1*	9.3*	27.9^δ^	10.1^δ^	26.6^§^	8.5^§^
Alcohol intake, %						
Never or rarely	22.4	23.3	34.5^δ^	30.7^δ^	14.3	15.4
Frequently	38.4	37.8	39.0^δ^	34.0^δ^	37.9	42.0
Daily	28.9	23.9	20.6^δ^	18.1^δ^	34.5	30.0
Not declared	10.3	15.0	5.9^δ^	17.2^δ^	13.3	12.6
Sedentary lifestyle (yes), %	14.8	10.5	14.7	10.7	14.8	10.2

*Differences between full sample in category 'death before the age of 80' *versus* full sample in category 'death after the age of 80'.

^δ^difference between women in category 'death before the age of 80' *versus* women in category 'death after the age of 80'.

^§^difference between men in category 'death before the age of 80' *versus* men in category 'death after the age of 80'.

Among women, the group that died before the age of 80, compared with those who died after this age, had more cancer and emotional/psychiatric problems, had less heart disease and hypertension as well as had less difficulty regarding instrumental activities of daily, lower prevalence of falls, lower care from mother and from father and greater overprotection from parents (Tables 2 and 3).

**Table 2:** Baseline clinical and functional characteristics who died before and after the age of 80 during 12-month follow-up.

	Full sample (N = 941)		Women (n = 445)		Men (n = 496)	
	Death before the age of 80 (n = 339)	Death after the age of 80 (n = 602)	Death before the age of 80 (n = 136)	Death after the age of 80 (n = 309)	Death before the age of 80 (n = 203)	Death after the age of 80 (n = 293)
**Health conditions**						
Stroke (yes), %	7.7	9.8	6.6	9.7	8.4	9.9
Heart disease (yes), %	32.7*	40.0*	29.4^δ^	41.7^δ^	35.0	38.2
Lung disease (yes), %	21.8*	15.6*	20.6	17.8	22.7^§^	13.3^§^
Cancer (yes), %	10.9*	7.1*	14.0^δ^	5.2^δ^	8.9	9.2
Osteoporosis (yes), %	10.0	14.1	19.1	22.3	3.9	5.5
Dementia (yes), %	0.7	0.6	0.7	0.6	0.5	0.7
Emotional/psychiatric problems (yes), %	7.1	3.7	12.5^δ^	3.9^δ^	3.4	3.4
Hypertension, (yes) %	49.9*	58.8*	49.3^δ^	64.1^δ^	50.2	51.5
Diabetes mellitus (yes), %	17.1	16.5	14.7	14.6	18.7	18.4
Fall in last 12 months (yes), %	26.3*	40.5*	27.2^δ^	45.6^δ^	25.6^§^	35.1^§^
Depressive symptoms (yes), %	17.4	17.3	19.1	23.6	16.3	10.6
**Functioning**						
Basic activities of daily living, (mean) (SD)	0.7 (1.3)	0.6 (1.1)	0.8 (1.5)	0.7 (1.2)	0.6 (1.2)	0.5 (1.0)
Instrumental activities of daily living, (mean) (SD)	0.5 (1.0)	0.6 (1.0)	0.5 (1.0)^δ^	0.8 (1.2)^δ^	0.5 (1.0)	0.4 (0.7)

*Differences between full sample in category 'death before the age of 80' *versus* full sample in category 'death after the age of 80'.

^δ^Difference between women in category 'death before the age of 80' *versus* women in category 'death after the age of 80';

^§^Difference between men in category 'death before the age of 80' *versus* men in category 'death after the age of 80'.

**Table 3:** Characteristics of adverse events in childhood or adolescence who died before and after the age of 80 during 12-month follow-up.

	Full sample (N = 941)		Women (n = 445)		Men (n = 496)	
	Death before the age of 80 (n = 339)	Death after the age of 80 (n = 602)	Death before the age of 80 (n = 136)	Death after the age of 80 (n = 309)	Death before the age of 80 (n = 339)	Death after the age of 80 (n = 602)
**Childhood or adolescence socioeconomic characteristics**						
Family structure, %						
Both biological parents	87.6*	92.5*	88.2	91.3	87.2^§^	93.9^§^
Mother and stepfather/father and stepmother	1,5	2.2	2.2	1.9	1.0^§^	2.4^§^
Only father or mother	7.3*	2.5*	6.6	2.9	7.9^§^	2.0^§^
Others	3.6	2.8	3.0	3.9	3.9	1.7
Occupation of head of household (low occupational class), %	37.8	36.2	30.1	37.9	42.9	34.5
Housing conditions, %						
5 amenities	2.7*	0.7*	2.9	0.7	2.4	0.7
4 amenities	42.2*	34.7*	42.6	38.2	41.9^§^	31.1^§^
3 amenities	11.2*	14.9*	10.3	16.8	11.8	13.0
2 amenities	9.7*	11.3*	11.8	8.7	8.4^§^	14.0^§^
1 amenity	26.8*	30.1*	26.5	27.5	27.1	32.7
0 amenities	7.4*	8.3*	5.9	8.1	8.4	8.5
Number of rooms in home (mean), (SD)	2.9 (1.0)	2.8 (0.8)	2.8 (0.8)	2.9 (1.0)	2.8 (0.7)	2.9 (1.0)
Number of residents in home (mean), (SD)	5.2 (2.2)	5.2 (1.9)	5.1 (2.2)	5.3 (1.9)	5.1 (2.1)	5.0 (1.8)
**Stress in childhood or adolescence**—**parental bonding instrument (PBI)**						
Care from mother (mean), (SD)	7.0 (1.7)	7.2 (1.6)	6.7 (2.0)^δ^	7.2 (1.6)^δ^	7.3 (1.4)	7.3 (1.5)
Care from father (mean), (SD)	6.5 (2.0)*	6.9 (1.7)*	6.3 (2.2)^δ^	7.0 (1.6)^δ^	6.7 (1.8)	6.8 (1.7)
Protection from mother (mean), (SD)	3.8 (2.1)*	3.4 (1.8)*	4.0 (2.3)^δ^	3.5 (1.8)^δ^	3.7 (2.0)	3.4 (1.8)
Protection from father (mean), (SD)	3.7 (1.9)*	3.3 (1.7)*	4.1 (2.1)^δ^	3.4 (1.7)^δ^	3.5 (1.8)	3.2 (1.7)
**Infectious disease in childhood or adolescence**						
Infectious disease (yes), %	90.2*	85.7*	94.8^δ^	89.0^δ^	87.2	82.2

*Differences between full sample in category 'death before the age of 80' *versus* full sample in category 'death after the age of 80'.

^δ^Difference between women in category 'death before the age of 80' *versus* women in category 'death after the age of 80';

^§^Difference between men in category 'death before the age of 80' *versus* men in category 'death after the age of 80'.

Among men, the group that died before the age of 80, compared with those who died after this age, had more lung disease, lower prevalence of falls and having lived only with the father or only with the mother (Tables 2 and 3).

### Risk factors for early mortality in both sexes

For both men and women, overprotection by the father in childhood or adolescence increased the risk of death before the age of 80 by 12% (OR 1.12; *p* = 0.04) for men and 22% (OR 1.22; *p* < 0.01) for women (Tables 4 and 5).

**Table 4:** Final model of factors associated with death before the age of 80 among women (n = 445) aged 50 years or older at baseline of ELSA study (2006) in 12-year follow-up period.

	Adjusted OR	95% CI
**Adverse events in childhood or adolescence**		
Occupation of head of household (low occupational class)	0.58	0.34–0.98*
PBI—care from mother	0.86	0.76–0.99*
PBI—protection from father	1.22	1.07–1.39*
Housing conditions, %		
5 amenities	1.00	
4 amenities	0.29	0.04–1.83
3 amenities	0.18	0.02–1.26
2 amenities	0.43	0.06–3.07
1 amenity	0.30	0.04–1.93
0 amenities	0.18	0.02–1.42
**Socioeconomic aspects**		
Marital status (single)	0.36	0.22–0.58**
Total household wealth (quintiles), %		
5th quintile (top)	1.00	
4th quintile	1.10	0.47–2.55
3rd quintile	0.66	0.28–1.53
2nd quintile	0.96	0.43–2.15
1st First quintile (lower)	0.82	0.35–1.88
Not declared	0.84	0.06–10.64
**Health behaviors**		
Smoking		
Non-smoker	1.00	
Ex-smoker	1.01	0.59–1.71
Smoker	3.69	1.90–7.18**
**Health conditions**		
Cancer (yes)	3.72	1.59–8.72*
Emotional/psychiatric problems (yes)	3.39	1.42–8.08*
Fall in last 12 months (yes)	0.38	0.23–0.63**

*OR* odds ratio, *CI* confidence interval; adjusted for current income and housing conditions in childhood.

*p < 0.05; **p < 0.001.

**Table 5:** Final model of factors associated with death before the age of 80 among men (n = 496) aged 50 years or older at baseline of ELSA study (2006) in 12-year follow-up period.

	Adjusted OR	95% CI
**Adverse events in childhood or adolescence**		
Family structure		
Both biological parents	1.00	
Mother and stepfather/father and stepmother	0.29	0.05–1.61
Only father or mother	3.79	1.35–10.67*
Others	2.17	0.63–7.50
PBI–protection from father	1.12	1.01–1.25*
Infectious disease (yes)	2.05	1.15–3.66*
Housing conditions, %		
5 amenities	1.00	
4 amenities	0.46	0.08–2.64
3 amenities	0.28	0.04–1.70
2 amenities	0.20	0.03–1.27
1 amenity	0.24	0.04–1.40
0 amenities	0.34	0.05–2.16
**Socioeconomic aspects**		
Marital status (single)	0.57	0.35–0.92*
Total household wealth (quintiles)		
Fifth quintile (top)	1.00	
Fourth quintile	1.26	0.66–2.41
Third quintile	1.56	0.81–3.00
Second quintile	2.40	1.17–4.91*
First quintile (lower)	2.13	1.04–4.34*
Not declared	2.42	0.43–13.54
**Health behaviors**		
Smoking		
Non-smoker	1.00	
Ex-smoker	0.90	0.54–1.47
Smoker	3.63	1.84–7.18**
**Health conditions**		
Fall in last 12 months (yes)	0.55	0.36–0.86*

*OR* odds ratio, *CI* confidence interval; adjusted for current income and housing conditions in childhood.

*p < 0.05; **p < 0.001.

### Risk factors for early mortality in women

However, the head of the household having a low occupation diminished the risk of death before 80 years by 42% (OR 0.58; *p* = 0.04), whereas having more care from the mother reduced the risk of premature death by 14% (OR 0.86; *p* = 0.03). Besides early life conditions, issues in adulthood, such as being a smoker, reporting cancer and emotional/psychiatric problems, increased the risk of death before 80 years by 269% (OR 3.69; *p* = 0.00), 272% (OR 3.72; *p* < 0.01) and 239% (OR 3.39; *p* < 0.01), respectively, and being single diminished the risk of death before 80 years by 64% (OR 0.36; *p* = 0.00) (Table 4).

### Risk factors for early mortality in men

For men, having lived with only one of the parents and having had an infectious disease in childhood or adolescence increased the risk of death before 80 years by 279% (OR 3.79; *p* = 0.01), and 105% (OR 2.05; *p* = 0.01), respectively. Regarding issues related to adulthood, having a low socioeconomic status increased the risk of death before 80 years by 113% (OR 2.13; *p* = 0.03) and being a smoker increased the risk by 263% (OR 3.63; *p* = 0.00) (Table 5).

### Comparisons between included and excluded individuals

In the analysis comparing those individuals included in the study to those excluded due to missing information, the included individuals were older, were mostly married, had a higher income, were more active and consumed more alcohol. Cancer, emotional/psychiatric and depressive symptoms were less prevalent among the included individuals, who also had fewer difficulties regarding instrumental activities of daily living than the excluded individuals. Regarding adverse events in childhood or adolescence, most included individuals who were raised by both biological parents, had higher scores regarding care from both the father and the mother and had lower more scores regarding protection from the father in comparison to the excluded individuals (Supplementary Tables S1 and S2).

## Discussion

The key findings of the present study indicate that associations between adverse events in childhood or adolescence and mortality under the age of 80 differ between older men and women. While living with only one parent and experiencing an infectious disease in childhood or adolescence were associated with a greater risk of mortality before the age of 80 among men, a high level of care from the mother and a low occupational class of the head of the household were protective factors for women. Interestingly, the only common factor in both sexes was an increased risk of death before the age of 80 among individuals who had an overprotective father.

Among the adverse events in childhood or adolescence, overprotection on the part of the father increased the risk of mortality before the age of 80 in both sexes, whereas a high level of care from the mother was a protective factor only for women. In a study involving 1964 English individuals between 65 and 79 years of age, Demakakos et al. found that overprotection and a low level of care from both parents increased the risk of mortality in a six-year follow-up period^5^. However, their analyses were not stratified by sex and there was no separate analysis of care and protection from the parents, as done in the present study, which enabled a better understanding of these associations.

Overprotection from the father in childhood or adolescence may exert a negative influence on the development of autonomy and on behavior throughout life^42^. As the father presents an authoritarian figure, this could increase the risk of developing psychological disorders, which could interfere with one’s quality of life and contribute to the increase in the risk of premature death^43^. Similar results are reported in a case–control study by Turgeon et al., who found that individuals with obsessive–compulsive disorder and panic syndrome had higher scores for overprotection by both parents in comparison to the healthy group^44^.

In addition to protection, care is another important mediator of repercussions throughout life^6^. The present findings indicate that a high level of care from the mother exerted a protective effect against mortality before the age of 80 only for women. Low levels of care can trigger stress in childhood or adolescence, favoring the development of depression and other mental health disorders that increase the risk of death^6^. An Australian group evaluated the association between parental style in childhood and the development of anxiety and depression in adulthood among 289 university students using the Parental Bonding Instrument (PBI). The researchers found that depression and anxiety were more prevalent among individuals with low levels of care from the mother compared to those with high levels of care^45^.

The fact that our results were significant only with regards to care from the mother and not the father may be explained by the favorable maternal bond, as most mothers stayed at home taking care of the family, while the fathers were working^46^. Moreover, it is possible that there is a difference between sexes in the impact of parenting. In the past, mothers spent more time with their children and daughters had stronger ties to their mothers than sons, as men were encouraged to leave home earlier^46^. Therefore, this difference may also explain the fact that we found a protective effect against mortality before the age of 80 only among women.

Regarding clinical characteristics, the report of an infectious disease in childhood or adolescence was a risk factor for mortality before the age of 80 among men in the present study. Blackwell et al. reported similar results investigating the effect of adverse events in childhood on the health of older American adults. They found that chronic diseases, which are highly associated with premature death, were more prevalent in both men and women who had episodes of infectious diseases in childhood or adolescence^47^.

One explanation for this phenomenon is that infectious processes in childhood or adolescence can increase inflammatory activity and, consequently, the level of inflammatory markers (C-reactive protein, interleukin-6, tumor necrosis factor and fibrinogen) at a time when organs and systems are in a development phase^48,49^. This, in turn, could result in a reduction in anti-inflammatory activity not only in childhood or adolescence but with effects that could last throughout life^48,49^. Therefore, the trigger of infectious diseases in childhood or adolescence may increase inflammatory activity permanently, triggering chronic diseases associated with events that can lead to premature death, such as stroke and acute myocardial infarction^48^.

However, the response mechanism to infectious processes seems to differ between men and women, suggesting the influence of sex hormones. In response to inflammation resulting from infection, there is a more accentuated increase in tumor necrosis factor alpha (TNF-α) in men, which may result in significantly lower levels of interleukin-10 (IL-10), a potent anti-inflammatory cytokine^50^. This pattern is inverted in women (lower TNF-α and higher IL-10 levels). Therefore, the different modulation of the cytokine network by sex hormones, with a predominance of anti-inflammatory mediators, may have a protective effect in women, resulting in a lower risk of premature death compared to men^50^.

The present results indicate that men who lived with only one of their parents in childhood or adolescence were at greater risk of death prior to 80 years of age. This finding can be corroborated by an 8-year longitudinal study involving 13,723 individuals, in which Kang et al. found that men and women who lived with only one parent in childhood were at greater risk of death than those who lived with both parents and the association was significantly stronger among men^51^.

The absence of a parent implies greater socioeconomic hardship as well as less emotional support during childhood or adolescence^52,53^. The consequences of this situation seem to be worse for men. There is available evidence that men, who are more introverted than women, have greater difficulty seeking support from others in adverse situations when one of the parents is not present^53,54^. Therefore, the absence a parent in childhood or adolescence may exert a stronger negative influence on men with consequences that affect health throughout life and could be reflected in a premature death^53^ which corroborates our findings.

The occupation of the head of the household is another important component of early socioeconomic status^8,52^. The fact that a low occupational class was a protective factor against mortality before the age of 80 among women disagrees with most studies that demonstrated that a low occupational class of the head of the household is a risk factor for a poor functional performance in adulthood^55^ and premature death^56^. The greater risk of negative outcomes among children of parents from a low occupational class is attributed to a poorer socioeconomic status and less time spent by parents with their children due to the higher workload^57^.

Despite the paucity of evidence on this topic, we may speculate that the parents of these individuals had a more affectionate relationship with their daughters despite the less contact time, resulting in closer relationships^57^. Moreover, daughters tend to be more connected to their family than sons, playing an important role in family relations, which could result in a sense of greater wellbeing within the home. As a result, triggers for adverse situations would be minimized^58^ and, consequently, leading to a lower risk of premature death.

Our findings showed that current conditions were also associated with a greater risk of dying before the age of 80 in both men and women. Some risk factors for mortality before the age of 80 are widely known in the literature, such as smoking^59^ and being single^60^ (for both sexes), a low income^61^ (only for men), cancer^62^ and emotional/psychiatric problems^63^ (for women).

The present study has several strengths. The strongest point is the use of a representative national sample of community-dwelling older English adults, which enabled selecting large groups of men and women who died before and after the age of 80. Moreover, we measured socioeconomic status, stressful parental experiences and infectious diseases in childhood or adolescence as well as current socioeconomic, behavioral and clinical characteristics, which enabled the determination of distinct risk factors for mortality before the age of 80 for both sexes.

This study has also limitations that should be acknowledged. We recognize that the evaluation of health status based on the presence or absence of a group of infectious diseases during childhood without discriminating each one of the diseases could be potentially a limitation. However, Monteverde et al.^3^ analyzed this condition in the same manner. Moreover, individuals who did not answer the life history questionnaire may not have done so due to having recall bias or possibly having a traumatic childhood or adolescence that they did not want to bring up, which may also be a source of bias, leading to an underestimation of the results of our associations. It was not possible to include individuals who died prior to 50 years of age, as the study involved individuals aged 50 years or older. Therefore, there could be some degree of survival bias, as adverse events may have led to death prior to reaching this age. Lastly, although the individuals excluded were younger and consumed less alcohol than those included, the excluded individuals had more psychiatric and emotional problems, lived more with only the father, only the mother or with other people in childhood or adolescence, had less care from the mother and father and more overprotection from the father. All this may have underestimated the associations we found between adverse events in childhood or adolescence and premature death.

## Conclusion

Worse socioeconomic status, infectious disease and bad parental relationship during childhood or adolescence are associated with the risk of premature death differently in men and women. Men seem to suffer more the consequences of adverse events in childhood or adolescence than women. Given the differences in the relationships of men and women with fathers and mothers in childhood or adolescence, overprotection on the part of the father increases the risk of premature death in both sexes, whereas a high level of care from the mother protects only women from this situation. Therefore, understanding the effects of adverse events in childhood or adolescence could assist in the creation of public policies directed at ensuring better care for children and adolescents and, ultimately, the promotion of healthy aging.

## Supplementary Information

Supplementary Tables.

## Data Availability

The data supporting the conclusions is all contained within the manuscript and supplementary information.
