# Supplementary material for: Gender differences in the association between adverse events in childhood or adolescence and the risk of premature mortality
Source: Sci Rep. 2022 Nov 9;12:19118. doi: 10.1038/s41598-022-23443-y (PMC9646814; doi:10.1038/s41598-022-23443-y)
Supplement: Supplementary file 1 — Supplementary Tables. [file 41598_2022_23443_MOESM1_ESM.pdf]

Gender differences in the association between adverse events in childhood or adolescence and the risk of premature mortality

Aline Fernanda de Souza, MSc[1], Roberta de Oliveira Máximo, PhD[1], Dayane Capra de Oliveira, PhD [1], Paula Camila Ramírez, PhD [1,2], Mariane Marques Luiz, MSc[1], Maicon Luis Bicigo Delinocente[3], MSc, Jair Licio Ferreira Santos, PhD[4], Andrew Steptoe, PhD[5], Cesar de Oliveira, PhD[5], Tiago da Silva Alexandre, PhD[1,3,5,6*]

Affiliations:

[1] Graduate Program in Physical Therapy, Federal University of São Carlos, São Carlos, Brazil.

[2] Escuela de Fisioterapia, Universidad Industrial de Santander, Bucaramanga, Colômbia

[3] Graduate Program in Gerontology, Federal University of São Carlos, São Carlos, Brazil.

[4] Department of Social Medicine, University of São Paulo, Ribeirão Preto, Brazil.

[5] Department of Epidemiology and Public Health, University College London, United Kingdom.

[6] Gerontology Department, Federal University of São Carlos, São Carlos, Brazil.

***Corresponding author:**

Department of Gerontology, Federal University of São Carlos, Rodovia Washington Luís, km 235, Sao Carlos, Sao Paulo 13565–905, E-mail: tiagoalexandre@ufscar.br / t.alexandre@ucl.ac.uk.

Supplementary material

Supplementary Table 1. Socioeconomic, behavioral, clinical and functional characteristics among individuals included in study and excluded due to missing data at baseline, ELSA study (2006).

	**Included (N = 941)**	**Excluded (N = 834)**
**Socioeconomic aspects**		
Age (mean), (SD)	82.9 (8.0)*	81.2 (10.9)*
Race (non-white), %	0.6	1.7
Marital status (single), %	45.7*	55.3*
Education years, %		
>13 years	21.9	23.0
12 – 13 years	18.7	18.5
0 – 11 years	59.4	58.5
Total household wealth (quintiles), %		
5th quintile (top)	14.9	13.1
4th quintile	20.3*	14.4*
3rd quintile	21.4	20.1
2nd quintile	19.9	20.5
1st First quintile (lower)	22.4*	29.9*
Not declared	1.1	2.0
**Health behaviors**		
Smoking, %		
Non-smoker	31.6	28.1
Ex-smoker	52.7	52.2
Smoker	15.7	19.7
Alcohol intake, %		
Never or rarely	23.0	22.5
Frequently	38.0*	27.6*
Daily	25.7	22.3
Not declared	13.3*	27.6*
Sedentary lifestyle (yes), %	12.0*	17.0*
**Health conditions**		
Stroke (yes), %	9.0	10.1
Heart disease (yes), %	37.4	35.4
Lung disease (yes), %	17.8	20.5
Cancer (yes), %	8.5*	8.4*
Osteoporosis (yes), %	12.6	11.0
Dementia (yes), %	0.6	1.4
Emotional/psychiatric problems (yes), %	4.9*	8.2*
Hypertension, (yes) %	55.1	54.5
Diabetes mellitus (yes), %	16.7	13.7
Fall in last 12 months (yes), %	35.4	39.5
Depressive symptoms (yes), %	17.3*	24.5*
**Functioning**		
Basic activities of daily living, (mean) (SD)	0.6 (1.2)	0.7 (1.2)
Instrumental activities of daily living, (mean) (SD)	0.6 (1.0)*	0.7 (1.2)*

Note. * Differences between included and excluded (p <0.05).

Supplementary Table 2. Adverse events in childhood or adolescence among individuals included in study and excluded due to missing data at baseline, ELSA study (2006).

	**Included (N = 941)**	**Excluded (N = 834)**
**Childhood or adolescence socioeconomic characteristics**		
Family structure, %		
Both biological parents	90.8*	78.3*
Mother and stepfather/father and stepmother	1.9	2.4
Only father or mother	4.2*	11.2*
Others	3.1*	8.1*
Occupation of head of household (low occupational class), %	36.8	40.5
Housing conditions, %		
5 amenities	1.4	1.9
4 amenities	37.4	38.9
3 amenities	13.6	13.6
2 amenities	10.7	9.1
1 amenity	28.9	25.9
0 amenities	8.0	10.6
Number of rooms in home (mean), (SD)	5.2 (2.0)	5.2 (2.0)
Number of residents in home (mean), (SD)	2.9 (0.9)	2.9 (0.9)
**Stress in childhood or adolescence – Parental Bonding Instrument (PBI)**		
Care from mother (mean), (SD)	7.2 (1.6)*	6.7 (2.0)*
Care from father (mean), (SD)	6.8 (1.8)*	6.3 (1.9)*
Protection from mother (mean), (SD)	3.6 (1.9)	3.9 (1.9)
Protection from father (mean), (SD)	3.5 (1.8)*	3.7 (1.8)*
**Infectious disease in childhood or adolescence**		
Infectious disease (yes), %	87.4	84.9

Note. * Differences between included and excluded (p <0.05).
